# Impact of Packing and Processing Technique on Mechanical Properties of Acrylic Denture Base Materials

**DOI:** 10.3390/ma8052093

**Published:** 2015-04-24

**Authors:** Touraj Nejatian, Farshid Sefat, Tony Johnson

**Affiliations:** 1 Dental Materials Unit, School of Dental Sciences, University of Newcastle, Newcastle upon Tyne NE2 4BW, UK; E-Mail: Touraj.Nejatian@newcastle.ac.uk; 2 School of Clinical Dentistry, University of Sheffield, Sheffield, South Yorkshire S10 2TA, UK; E-Mail: a.johnson@sheffield.ac.uk; 3 Department of Biomedical Engineering, School of Engineering, King Faisal University, Al-Hofuf, Al-Ahsa 31982, Saudi Arabia; 4 Tissue Engineering Group, Department of Chemistry, Chemical Biology and Biomedical Engineering, Stevens Institute of Technology, Hoboken, NJ 07030, USA

**Keywords:** polymethylmethacrylate (PMMA), acrylic denture, mechanical properties

## Abstract

The fracture resistance of polymethylmethacrylate (PMMA) as the most popular denture base material is not satisfactory. Different factors can be involved in denture fracture. Among them, flexural fatigue and impact are the most common failure mechanisms of an acrylic denture base. It has been shown that there is a correlation between the static strength and fatigue life of composite resins. Therefore, the transverse strength of the denture base materials can be an important indicator of their service life. In order to improve the fracture resistance of PMMA, extensive studies have been carried out; however, only a few promising results were achieved, which are limited to some mechanical properties of PMMA at the cost of other properties. This study aimed at optimizing the packing and processing condition of heat-cured PMMA as a denture base resin in order to improve its biaxial flexural strength (BFS). The results showed that the plain type of resin with a powder/monomer ratio of 2.5:1 or less, packed conventionally and cured in a water bath for 2 h at 95 °C provides the highest BFS. Also, it was found that the performance of the dry heat processor is inconsistent with the number of flasks being loaded.

## 1. Introduction

Polymethyl methacrylate (PMMA) has been the most popular material for construction of dentures since the 1930s due to many advantages, including good aesthetics, accurate fit, stability in the oral environment, easy laboratory and clinical manipulation, and inexpensive equipment. However, the fracture resistance of PMMA is not satisfactory [1,2]. According to a survey, two-third of dentures had broken within three years of their provision [3]. Darbar *et al.* 1994, reported that 33% of the repairs carried out by three laboratories were due to debonded/detached teeth; 29% were due to midline fractures which were most common in the upper dentures; and the rest (38%) were other types of fracture. Denture fracture is a multifactorial phenomenon, and even strengthening measures could not efficiently prevent denture fracture [4].

Flexural fatigue and impact fracture have been implicated as a mechanism of denture fracture. Therefore, the transverse strength of the denture base materials can be an important indicator of their performance [5,6]. To reduce the denture fracture incidence, four different aspects can be considered based on the etiology of the fracture: (1) retaining the mechanical characteristics through corrective surgery of anatomic abnormalities such as high frenum and palatal torus, improving denture fit and balanced occlusion; (2) optimizing chemical structure through modifying packing and processing techniques [7]; (3) improving adhesion between acrylic teeth and the denture base resin [8]; (4) altering the composition either chemically by, for example, changing brittle polymers to a high impact polymer through the addition of rubber particles [9]; or making physical alterations that in this case incorporate materials into PMMA such as fibers, metal inserts, and particles [10]. An additional method is using alternative materials such as polyamides, epoxy resin, polystyrene vinyl acrylic, polycarbonate, polyurethane and nylon [11,12,13].

Despite a great deal of effort, the fracture PMMA denture base material is still a matter of concern for both dentists and patients [14]. Few promising results, limited to some mechanical properties of PMMA, have often been achieved at the cost of other properties [10,15]. This study was designed to evaluate the effect of processing methods, packing techniques, and the type of resin on the fracture resistance of PMMA as a denture base material. The study design is based on a multi-factorial experiment to evaluate the influence of five factors; resin type, powder-toliquid ratio, packing technique, processing method and curing time on flexural strength of the resin. In addition, the performance of acrylic processors, dry heat oven and water bath, were assessed and compared. The effect of extruder angle on mechanical properties of PMMA packed using an injection technique was also studied. In this study, a biaxial flexural strength (BFS) test was used to measure bending strength of PMMA. BFS and four-point bend tests have been employed to study PMMA and resin composites [16,17] as they are known to be better indicators of pure bending strength in comparison with the three-point bend test due to less shear stress generated in the specimen [16,18,19,20]. In the case of the BFS test, the specimen is supported near to its periphery and, as the stress rises, far from the edge that makes the test less sensitive to edge defects, which inevitably occur during the preparation. Therefore, it provides less scatter in the data [18,21]. Also, easy sample preparation and its size match to the clinical application make the BFS test a reproducible test to measure the flexural strength of these materials [18,22].

## 2. Results and Discussion

### 2.1. Effect of the Resin Type (Factor A)

**Figure 1:**
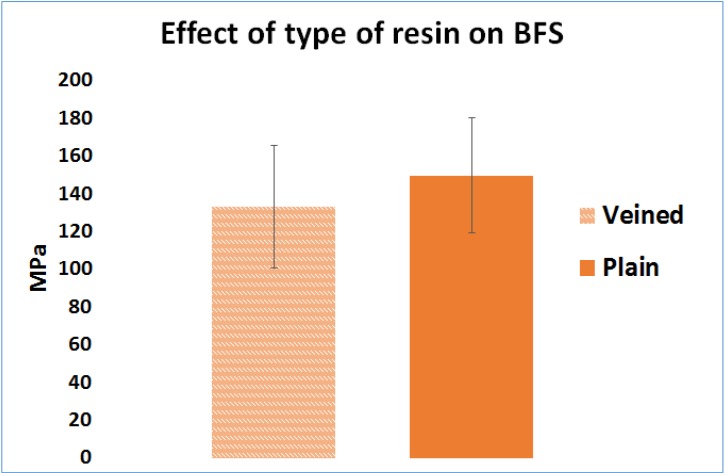
Effect of resin type on Biaxial Flexural Strength.

The mean BFS of the samples, made from plain PMMA (149 MPa, SD = 30), were higher than the veined type (133 MPa, SD = 32), and the difference was statistically significant (*n* = 300, *p* < 0.05) (Figure 1). 

### 2.2. Effect of Packing Method on Biaxial Flexural Strength (Factor B)

**Figure 2:**
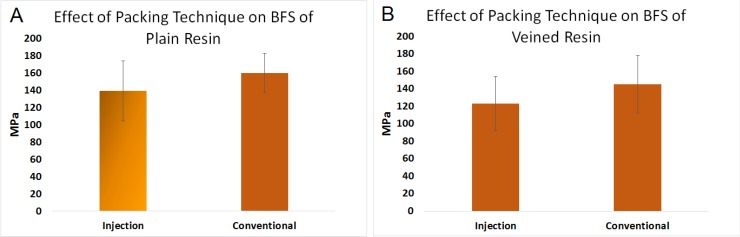
Effect of packing method on BFS of (**A**) plain polymethylmethacrylate (PMMA); and (**B**) veined PMMA for conventional and injection.

Figure 2A,B represent the data for the effect of the packing method on the BFS of PMMA. The mean BFS of conventionally packed plain (159 MPa, SD = 22), and veined PMMA (145 MPa, SD = 32), discs was greater than the injection-packed ones (139 MPa, SD = 34 and 123 MPa, SD = 31 respectively), and the differences were statistically significant (*n* = 150, *p* < 0.05).

### 2.3. Effect of Powder-to-Liquid Ratio on BFS (Factor C)

**Figure 3:**
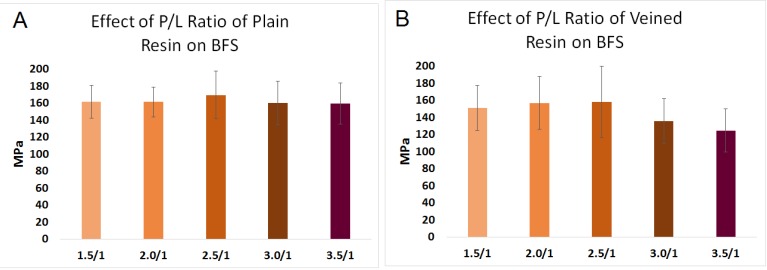
Effect of power/liquid ratio on BFS of (**A**) plain PMMA; and (**B**) veined PMMA for both plain and veined PMMA.

The data for the effect of the powder-to-liquid ratio on the BFS are illustrated in Figure 3A,B. Increasing the powder/liquid ratio of plain resin from 1.5/l to 3.5/l had no effect on the BFS of the resin (161 MPa, SD = 18 and 161 MPa, SD = 17 and 169 MPa, SD = 27 and159 MPa, SD = 25 and 159 MPa, SD = 24, respectively), (*n* = 30, *p* > 0.05). Similarly, changing powder/liquid ratio of veined resin from 1.5/l to 2.5/l had no significant effect on the BFS of the resin (150 MPa, SD = 26 and 156 MPa, SD = 30 and 158 MPa, SD = 41, respectively) (*p* > 0.05). Raising the powder ratio to 3 and 3.5 with respect to the monomer reduced the mean strength (135 MPa, SD = 26 and 124 MPa, SD = 25, respectively) compared to the 2.5/l ratio. The difference was statistically significant (*n* = 30, *p* < 0.05).

### 2.4. Effect of Curing Time in Water Bath (Factor E)

**Figure 4:**
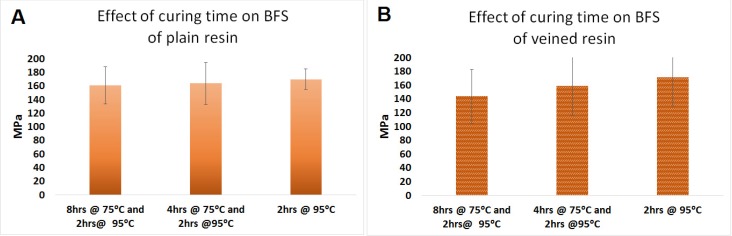
Effect of curing time in water bath on BFS of (**A**) plain and (**B**) veined PMMA.

The data for the effect of the curing time in the water bath on the BFS are presented in Figure 4A,B. Changing the curing time in the water bath from 8 h at 75 °C and 2 h at 95 °C to 4 h at 75 °C and 2 h at 95 °C and then 2 h at 95 °C did not have any effect on the mean BFS of the plain (170 MPa, SD = 15 and 164 MPa, SD = 31 and 161 MPa, SD = 27, respectively) and veined PMMA (172 MPa, SD = 42 and 159 MPa, SD = 42 and 144 MPa, SD = 39, respectively) (*n* = 10, *p* > 0.05) (Figure 4).

### 2.5. Effect of Extruder Angle on Mechanical Properties of PMMA

#### 2.5.1. Effect of Extruder Angle on BFS of PMMA

**Figure 5:**
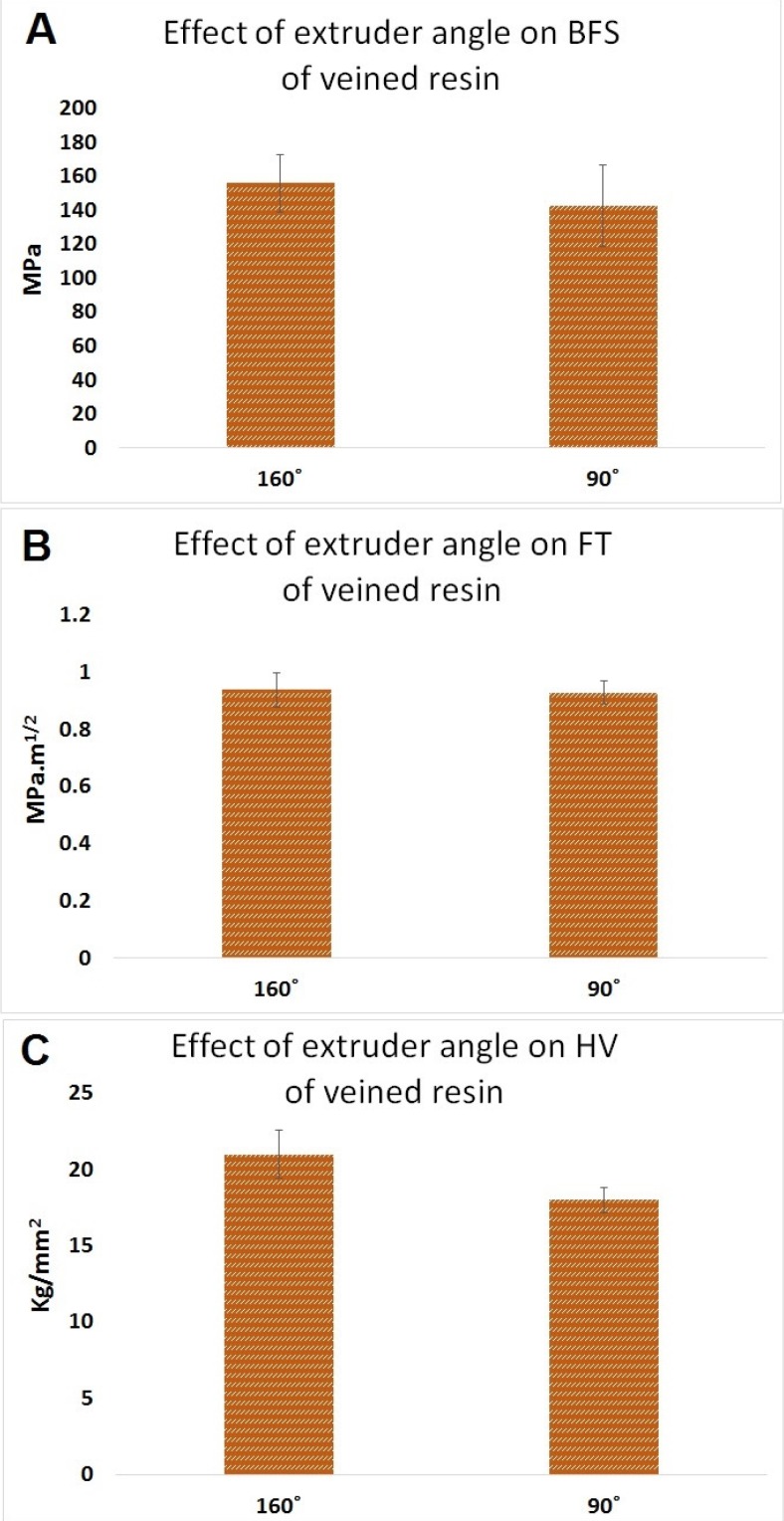
Effect of extruder angle on (**A**) BFS; (**B**) fracture toughness (FT); and (**C**) HV of veined PMMA. VIm W (veined type of resin packed using modified extruder, 160°, cured in the water bath), VIW (veined type of resin packed using standard extruder, 90°, cured in the water bath).

The data for the effect of extruder angle on the BFS of PMMA are shown in Figure 5A. Altering the extruder angle from 90° to 160° did not improve the mean BFS of veined type of the resin (156 MPa, SD = 17 and 143 MPa, SD = 24, respectively) (*n* = 10, *p* > 0.05).

#### 2.5.2. Effect of Extruder Angle on Fracture Toughness of PMMA

The data for the effect of extruder angle on fracture toughness of PMMA are shown in Figure 4B; altering the extruder angle from 90° to 160° did not improve the mean fracture toughness of veined type of the resin (0.94 MPa·m^1/2^, SD = 0.06 and 0.93 MPa·m^1/2^, SD = 0.04, respectively) (*n* = 30, *p* > 0.05).

#### 2.5.3. Effect of Extruder Angle on Hardness of PMMA

The data for the effect of extruder angle on Vicker’s hardness, HV, of PMMA shown in Figure 4C. Altering the extruder angle from 90° to 160° improved the mean Vicker’s hardness of veined type of the resin, but the difference was statistically significant (21 kg/mm^2^, SD = 1.6 and 18.4 kg/mm^2^, SD = 0.8, respectively) (*n* = 20, *p* < 0.05).

### 2.6. Analysis of Temperature within Curing Baths; Inside and Outside Curing Resin

**Figure 6:**
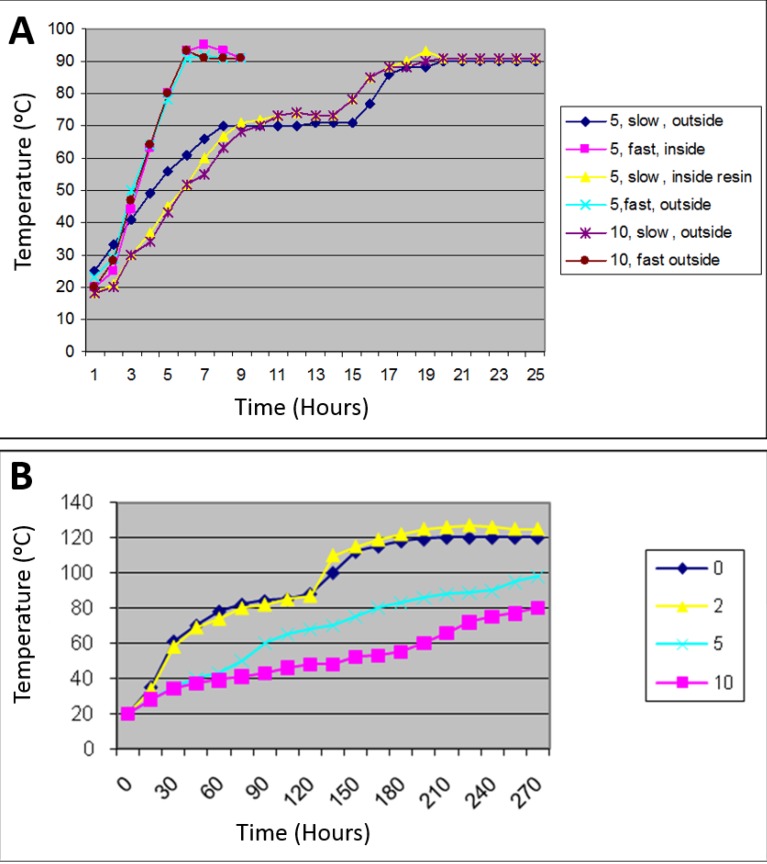

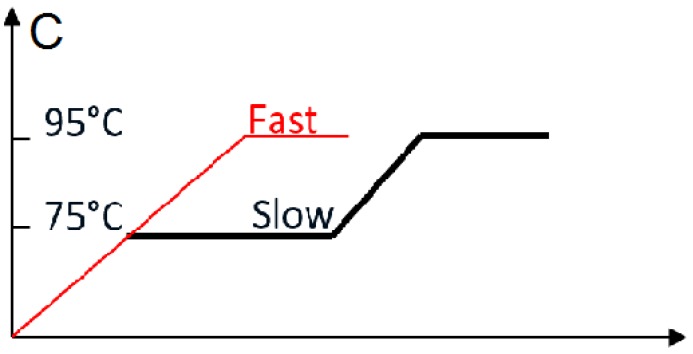
Pattern of temperature in (**A**) water bath loaded with 5 and 10 flasks and cure under slow (4 h 75 °C + 2 h 95 °C) and fast (2 h 95 °C) processing methods was measured; (**B**) Pattern of temperature inside the curing resin while water bath loaded with 5 flasks was measured; (**C**) Pattern of temperature in dry heat oven loaded with 0, 2, 5 and 10 flasks.

The overall data for the effect of the resin type on the BFS are presented in Figure 6A.

Analysis of temperature climb and hold within the curing chambers showed a consistent performance for the water bath irrespective of the number of flasks being cured. It was also found that the pattern of the temperature change inside the resin during curing is identical to that seen outside the resin even with different numbers of flasks being loaded (Figure 6A). In comparison with the standard setting (Figure 6C), the dry heat processor showed inconsistent results with flask numbers having a major effect on the rate of climb and holding temperature. In other words, the more flasks that were loaded, the slower the temperature elevated. When the oven is empty or loaded with up to two flasks, the chamber reached 120 °C, which is beyond the optimum curing temperature of 95 °C. Conversely, when loaded with 10 flasks, the oven could only reach a temperature of 80 °C, which is less than the required curing temperature. The accepted result was achieved when the oven was loaded with five flasks. (Figure 6B).

The standard setting of two curing methods in the dry heat processor: (1) the fast curing method in which temperature starts increasing steadily from 0 to 95 °C and then remains constant for a period of time (the time is adjustable); (2) The slow curing method in which temperature increases from 0 to 75 °C and remains constant for a period of time (the time is adjustable) then climbs up again and remains constant at 95 °C for a period of time (the time is adjustable).

### 2.7. Discussion

This study was essentially designed to evaluate the effect of resin type and its processing and packing variables on the BFS of heat-cure denture base PMMA. This was to optimize the condition to improve the BFS of PMMA, which may help increase the fracture resistance of denture base. Evaluating the temperature within the curing chambers during processing revealed that the number of flasks loaded in a dry heat oven seriously affects the performance of the processor. However, there was no difference between thermal changes inside and outside the curing resin, within the curing chamber. No evidence was found to study and compare the performance of processing. Due to the inconsistency of temperature and rate of climb within the dry heat processor, this curing method was not used for the main body of the work.

**Figure 7:**
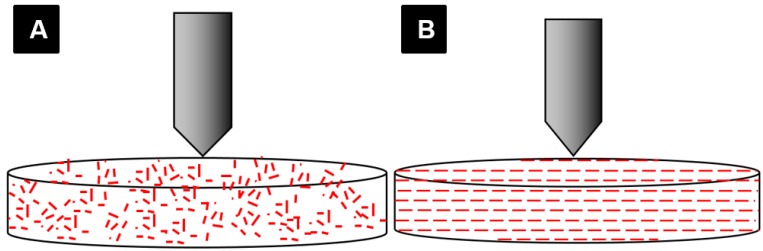
Schematic feature of fibers oriented (**A**) randomly (produced by right angle extruder); (**B**) organized perpendicular to direction of load (produced by 160° extruder).

Plain resin showed higher BFS results than the veined resin, which may be caused by weak bonding between the fibers and matrix. In this case, fibers may act as inclusions that weaken the resin. This might possibly be investigated by scanning electron microscopy. When considering the packing method, the conventionally packed plain and veined resins showed higher BFS results than the injection-packed ones, which may be because of disruption in the polymer chain formation due to the stretch caused by the turbulence effect that occurs inside the standard extruder syringe during the injection (Figure 11A). This can result in short and less cross-linked polymer chains, which is related to low strength of the resin. By altering the extruder angle from 90° to 160°, the mechanical properties of the resin were not improved. However, the standard deviation was decreased, possibly due to uniform flow and orientation of the fibers in the direction of injection and a reduced turbulence effect during the injection (Figure 7). Hardness value (HV) is directly related to elastic modulus and the resistance of material against plastic deformation, whereas BFS and FT are representative of resistance of the material against fracture. In other words, they are representative of two different types of properties of the material, which are not necessarily correlated. Unlike BFS and FT, improvement of HV was statistically significant which could be due to the concentration of the fibers on the superficial layer of the specimens. According to anecdotal evidence, this may happen because of marginalization of the fiber due to the friction of the resin with the internal surface of the mold during injection. As PMMA shows brittle behavior against impact forces, it is considered as a notch-sensitive material. Poor hardness value makes the material easily scratched by food or negligence, and the resulting micro-cracks and scratches can compromise the fracture resistance of the material. This part of the study was unique in the context of dental materials. To confirm the aforementioned hypotheses, further chemical analyses and electron microscopic studies would need to be carried out. Previous studies on the effects of packing method on flexural strength showed inconsistent results, probably due to different testing methods, sample size, and shape [23,24].

Although powder/liquid ratio of equivalent to 2:1 (volume) was recommended by the manufacturer, in this study, at least in the case of veined resin, increasing the powder/liquid ratio up to 2.5:1 increased the strength of the resin, but beyond that ratio, the strength of the resin was decreased. Therefore, using powder/liquid of 2.5:1 seems to be recommendable. No specific study has been found to investigate the optimal powder/liquid ratio of the resin; however, Dogan *et al.* [25] stated that excess monomer has a deteriorating effect on mechanical characteristics of the resin.

Reducing the delay time at 75 °C from 8 to 4 and 0 hours has no significant effect on the BFS of the resin, but reducing the curing time to 1 hour at 95 °C resulted in a soft specimen, which was deformed due to the load and could not be tested. Softness of the specimens was probably due to the incomplete setting reaction and retaining excessive amount of residual monomer, which was retained in the material [26]. Therefore, in order to cure the resin, at least 2 h at 95 °C is required.

In previous studies, no evidence was found to show the effect of elimination of the delay time on the strength of PMMA. However, some of them reported that increasing the curing time from 30 to 60 min not only improves the mechanical properties of the resin [25,27], but also decreases the residual monomer in the cured PMMA. This evidence suggests that raising curing time to 2 h at 95 °C is required to achieve an optimum BFS of PMMA. However, extended curing time does not have any extra benefit. In addition, the curing time could not be extended beyond 2 hours due to the limitation in the processor time adjustability. Hayhurst and Johnson (2004) in a similar study investigated the effect of the type of resin, packing, and processing variables on the BFS of PMMA, and they found no significant improvement effect on the BFS of cured PMMA [28]. The sample size of that study, it should be noted, was small and may not have been enough to show some of the differences.

## 3. Experimental Section

### 3.1. Materials

Plain and veined types of PMMA as a cadmium-free co-polymer (Oracryl heat cure denture material, Bracon, Etchingham, West Sussex, UK), base plate wax (Cavex, Cavex Holland BV, 2003 RW Haarlem, The Netherlands), plaster coating solution (Cold Mould Seal, Metrodent Ltd., Paddock, UK), yellow soft paraffin (Ecolab Ltd., Leeds, UK) were used in this study.

### 3.2. Sample Preparation

Samples were prepared in order to evaluate resin type, processing, and packing factors. In the first stage, five processing variables were considered, including: (1) type of the resin (plain and veined shown by “P” and “V”); (2) five different powder-to-liquid ratios by volume (1.5:1, 2:1, 2.5:1, 3:1, 3.5 represented by numbers 1–5); (3) two packing techniques (conventional and injection shown by “C” and “I”) (4) two processing methods (dry heat and water bath presented by “D” and “W”); 5) and finally three different curing times including 4 h at 75 °C and 2 h at 95 °C (as a usual curing time), 8 h at 75 °C and 2 h at 95 °C (as an upper extreme) and 2 h at 95 °C as the lowest curing time. Table 1 is a multifactorial test table designed to evaluate the effect of the mentioned factors.

**Table 1:** Multifactorial test table was designed to evaluate the effect of five variables on the BFS of PMMA.

Multifactorial test to evaluate the effect of five variables on the BFS of PMMA																							
				**A**																			
				2 (n = 300)										1 (n = 300)									
				**B**										**B**									
				1 (n = 150)					2 (n = 300)					1 (n = 150)					2 (n = 150)				
				**C**					**C**					**C**					**C**				
				(n = 30)					(n = 30)					(n = 30)					(n = 30)				
				1	2	3	4	5	1	2	3	4	5	1	2	3	4	5	1	2	3	4	5
**D**	1	**E**	1																				
			2																				
			3																				
	2	**E**	1																				
			2																				
			3																				

### 3.3. Factor Codes

Each factor given a code in this study, including types of resin (Factor A: veined 1, plain 2), packing method (Factor B: conventional 1, injection 2), powder/ liquid ratio (Factor C: (Cell No. 1 (1.50:1), Cell No. 2 (2: 1), Cell No.3 (2.50:1), Cell No. 4 (3:1), Cell No. 5 (3.50:1), processing method (Factor D: water bath 1, dry heat 2) and, finally, curing times (Factor E: a) 2 h at 95 °C; b) 4 h at 75 °C and 2 h at 95 °C; 3) 8 h at 75 °C and 2 h at 95 °C). To assess the fracture resistance of the resin, the biaxial flexural strength (BFS) of the specimens was considered. The performance of the curing devices, changing temperature inside the resin during the curing procedure and the effect of changing extruder angle of injection machine, were also assessed.

### 3.4. Wax Discs’ Preparation

**Figure 8:**
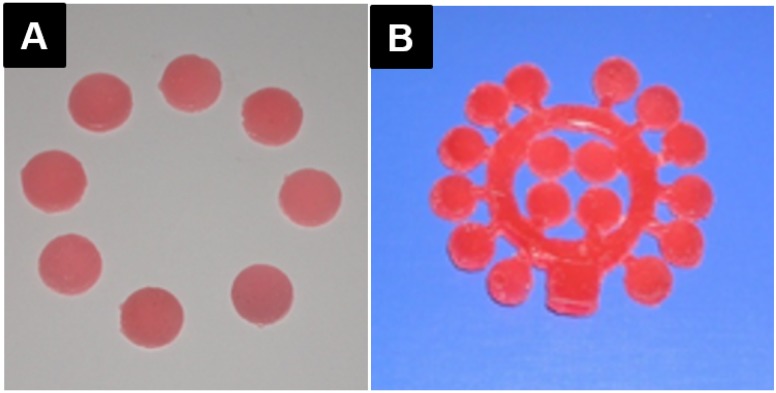
(**A**) Single-wax discs; (**B**) Tree-shaped arranged wax discs.

Single discs (Figure 8A) were prepared using a rubber mold; a tree-shaped 15-disc rubber mold was designed and made to prepare wax discs (Figure 8B) for the conventional- and injection-packing methods, respectively. Melted wax was poured into the rubber mold, left to cool, and then removed.

### 3.5. Flasking and Packing the Wax Discs

#### 3.5.1. Conventional Technique (Factor B1)

**Figure 9:**
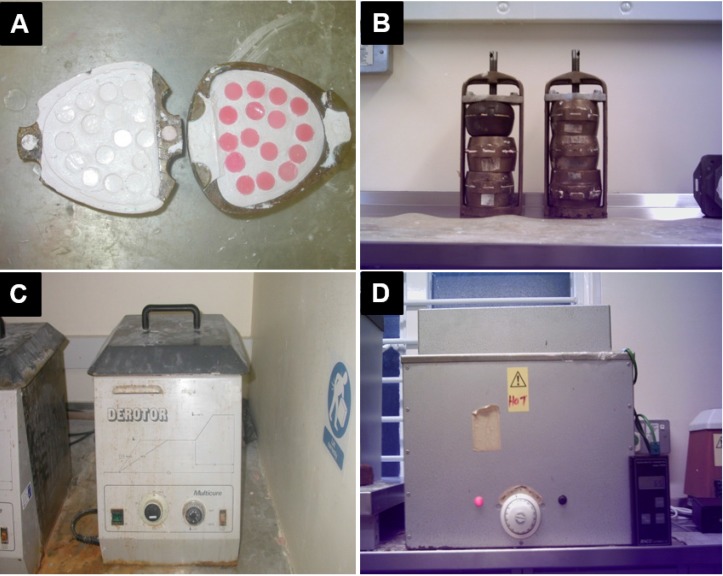
(**A**) Conventional method of flasking discs; (**B**) Conventionally packed and clamped flasks; (**C**) water bath (**D**) dry heat oven.

Plaster of Paris was mixed with water and poured inside one half of a two-part brass flask and then 15 prepared wax discs—with a 12 mm diameter and 2 mm thick—were inserted on the surface of the plaster. After initial setting of the plaster, all exposed disc surfaces were cleaned of plaster using water. Once the plaster was set, it was then lubricated with yellow soft paraffin, the flask was then filled with plaster of Paris and turned onto the first half, pushed together, and left to set completely (Figure 9A).

The flask was subsequently placed in the boiling water to remove the wax. After opening, the remaining wax was washed out using boiling water (Figure 9B). The mold surface was dried and painted with plaster coating solution. Two types of denture base acrylic resin powder, PMMA, plain and veined (Factor A), with five powder-to-liquid ratios 1.5:1, 2:1, 2.5:1, 3:1, 3.5:1 (Factor C) were mixed in a rubber bowl and left covered until ready to pack. The mixture was packed in the mold, pressed, and clamped conventionally (Figure 9B).

The clamped flask was put either in the water bath (Derotor water curing bath Quayle Dental Manufacturing CO. Ltd., Worthing, West Sussex, UK) (Figure 9C) or in the dry heat processor (Ditton dry acrylic processor, Chaperlin and Jacobs, Sutton, Surrey) (Figure 9D) (Factor D) and was cured with different curing times: (1) 8 h at 75 °C and 2 h at 95 °C; (2) 4 h at 75 °C and 2 h at 95 °C; (3) 2 h at 95 °C (Factor E).

#### 3.5.2. Injection Technique (Factor B2)

**Figure 10:**
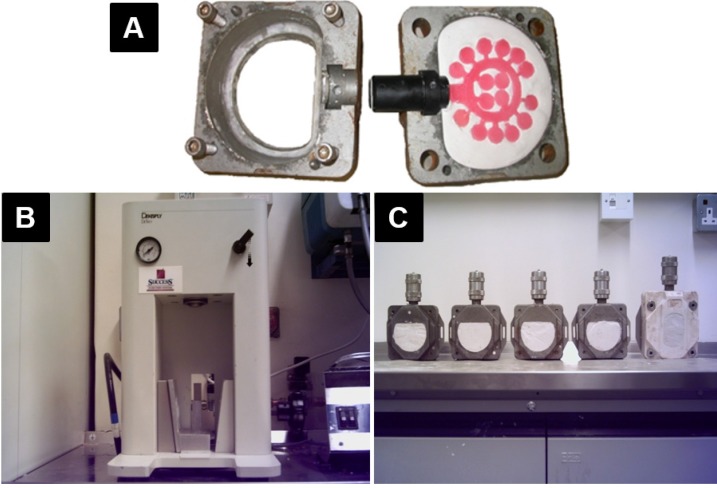
(**A**) Tree-shaped wax pattern invested in the flask with a plastic dummy; (**B**) Success Injection System™ (DENTSPLY International Inc., York, PA, USA); (**C**) Five injection-packed flasks.

The two halves of the injection flasks were lubricated internally. A plastic investing dummy was located on the opening of the flask (Figure 10A). The rest of the investing was identical to the conventional technique which was described previously. The plastic investing dummy was replaced with the metal injection nozzle and the two halves of the flask screwed together. The acrylic mixture (mixed as described previously for the conventional technique) was placed into a syringe which was installed on the nozzle and fixed in the injection machine (Success Injection System™, Dentsply DeTrey, Dreieich, Germany) (Figure 10B). The resin was injected under an atmosphere pressure of 4 into the flask and the pressure was maintained for 5 min.

After removing the syringe, a pressure-maintaining device was screwed on the nozzle to keep the pressure constant during the processing (Figure 10C). The resin was then processed in both the water and dry heat bath as described previously for the conventional technique.

### 3.6. De-Flasking and Preparing the Discs

After processing, the flasks were left to cool at room temperature and then opened up before removing the discs from the plaster. In the case of the injection technique, the discs were cut from the tree-shaped resin template and all excess resin “flash” trimmed using a hand piece and tungsten carbide bur. Finally, the discs were sandpapered with 600 µm silicon carbide sandpaper to achieve a completely flat surface and uniform thickness (±0.2 mm). The finished discs were stored at 37 °C for 48 ± 2 h in tap water before testing to comply with ISO standard (BS EN ISO 1567: 2002).

### 3.7. Modification of the Injection-Packing Technique

**Figure 11:**
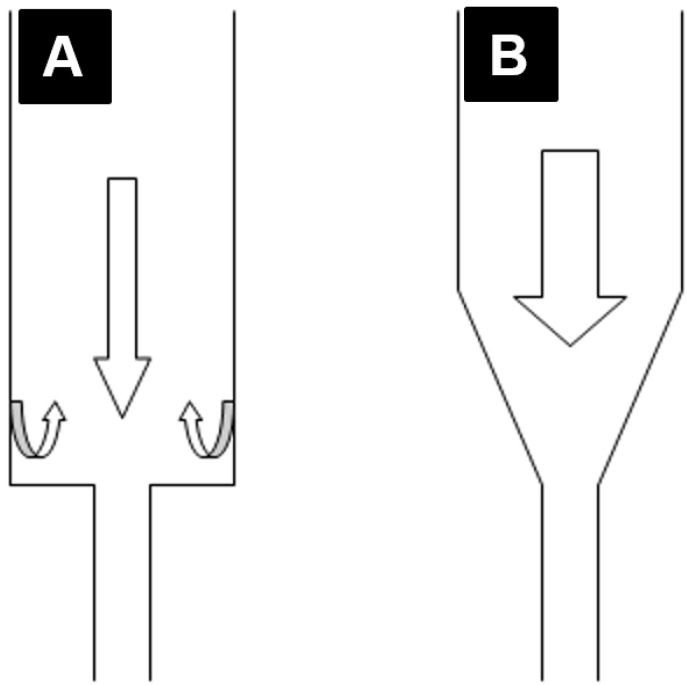
(**A**) Right angle, 90°, extruder; (**B**) modified angle, 160°, extruder.

Based on anecdotal evidence, the turbulence effect in the injection-molding system is a common problem in polymer engineering, which has been dealt with by altering the extruder angle. The old prefabricated extruder with a 90° angle was suspected of generating turbulence and stretch of the resin inside the extruder. An extruder with a modified angle of 160° was designed to reduce the probable deteriorating effect of the right angle extruder on the resin. Veined resin was prepared under optimal conditions and packed using the modified extruder. Biaxial flexural strength, Vickers hardness, and micro indentation fracture toughness tests were carried out on the samples, and the results were compared with those achieved using the standard right angle (Figure 11).

**Figure 12:**
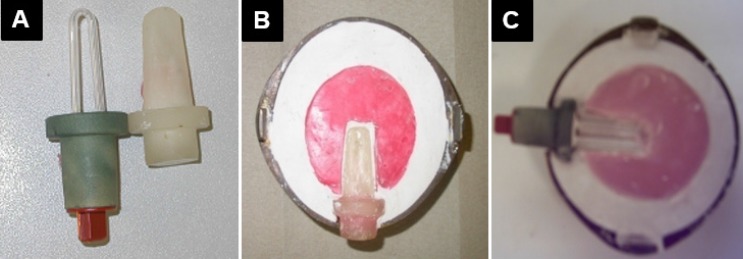
(**A**) Duplicated thermocouple head; (**B**) Investing the duplicated dummy; (**C**) Thermocouple head located inside the resin.

Due to the inconsistency of the results produced by the dry heat oven, the performance of the processors was suspected; an assessment of these processors was therefore undertaken. Water bath and dry heat oven were loaded with varying numbers of flasks to see the effect of flask numbers on the temperature and rate of climb. The thermal reaction inside the curing resin was also measured and compared with the internal temperature of curing chambers. This was carried out by drilling a hole in the middle of the flask into which a thermocouple (Jenco Electronics Ltd., Taipei, Taiwan, model 7001H) was placed to be in contact with the curing resin inside the flask (Figure 12A–C).

### 3.8. Evaluation of the Performance of the Processors

**Figure 13:**
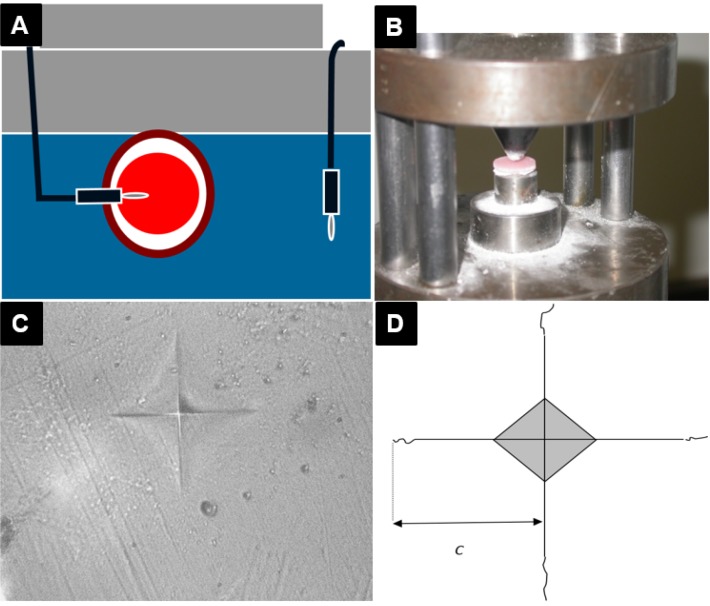
(**A**) Schematic of temperature measurement inside the resin and water bath; (**B**) Biaxial flexural strength (BFS) testing (ball on ring) using a tensile tester; (**C**) Microindentation Vickers hardness tests; (**D**) Crack length, *c*, on microindentation.

During processing, temperature readings were recorded every 15 min. The trend of the climb and hold patterns inside the curing chambers and acrylic resin was recorded using a Microcomputer Thermometer (Figure 13A). To evaluate the temperature within the curing bath, the tip of the thermometer was suspended inside the curing chambers. The temperature was recorded with no flasks in the processors and with varying numbers of flask inside the processors.

### 3.9. Biaxial Flexural Strength Testing

In this experiment, a sample size of 10 was calculated based on power = 0.85, Sigma = 20, Alpha = 0.05 and Maximum Difference = 30. Ten discs out of fifteen in each batch were selected based on having uniform thickness and no visual defect or porosity. The thickness of each disc was measured at three points with a digital micrometer (Quickmini, Mitutoyo Corp, Kawasaki, Japan) and the average thickness was calculated for each disc (±0.01 mm). A tensile-testing machine (Lloyd 2000R universal testing machine, Lloyd instrument Ltd., Fareham, Hants, UK) was employed to determine BFS of the discs. The discs were centrally placed onto an “O” ring and a ball tip instrument was used to apply load on the central point of the discs at a crosshead speed of 1 mm/min (Figure 13B). The BFS of the specimens was calculated using the following equation (Equation (1)).

(1) σmax=Ph2[0.606loge(ah)+1.13]

where σ_max_ is the maximum biaxial flexural strength; *P* is the load to fracture; *a* is the radius of the knife-edged support (O ring) and *h* is the sample thickness [29].

In order to measure the hardness of the composites, two discs were prepared for each composition and sandpapered with 240, 600 and 1200 grades of silicon carbide paper and then polished using 6 and 1 µm polycrystalline diamond abrasives (Buehler) to achieve a well-polished surface and even thickness (±0.1 mm). The discs were stored in water for 24 h at 37 °C. Ten Vickers indentations of 25 gf by microindenter (Mitutoyo hardness testing, Mitutoyo Ltd., Painesville, OH, USA) (Figure 13C) were applied on one of each of the disc surfaces and the hardness of the surfaces was calculated. To determine the microindentation fracture toughness [30], 30 indentations of 500 gf were carried out and the length of the cracks (2c) were measured by a Polyvar camera microscope (Reichert Polyvar Met Microscope, Wien, Austria) (Figure 13D). Using the average length of the radial cracks (*c*) and the following Evans and Charles equation (Equation (2)) [31], the fracture toughness of the composites was calculated:

*Klc* = 0.0752 *P*/*c*^3/2^ (2)

### 3.10. Data Analysis

The data were analyzed using one-way ANOVA and Tukey comparison at the 95% confidence level (*p* = 0.05) (Minitab release 13.1). 

## 4. Conclusions

This research was undertaken for evaluating the effect of type of resin, packing, and processing variables on the BFS of PMMA as a denture base resin. According to the results, the maximum biaxial flexural strength is achieved when the plain type of resin with a powder/liquid ratio of 2.5:1 was packed conventionally and cured in a water bath. The type of resin, packing procedure, and processing variables can have a significant effect on the BFS of PMMA. Based on the data presented, it can be concluded that plain resin is preferred over veined resin; a powder/liquid ratio between 1.5:1 and 2.5:1 can be used; conventional packing is preferred over injection molding; a water bath should be used instead of a dry heat oven; and, finally, a curing time of two hours at 95 °C is the optimum. 

## References

[1] Jagger D.C., Harrison A., Jandt K.D. (1999). The reinforcement of dentures. J. Oral Rehabil..

[2] John J., Gangadhar S.A., Shah I. (2001). Flexural strength of heat-polymerized polymethyl methacrylate denture resin reinforced with glass, aramid, or nylon fibers. J. Prosthet. Dent..

[3] Johnston E.P., Smith D.E. (1981). Flexural fatigue of 10 commonly used denture base resins. J. Prosthet. Dent..

[4] Darbar U.R., Huggett R., Harrison A. (1994). Denture fracture—A survey. Br. Dent. J..

[5] Vallittu P.K., Alakuijala P., Lassila V.P., Lappalainen V. (1994). *In vitro* fatigue fracture of an acrylic resin-based partial denture: An exploratory study. J. Prosthet. Dent..

[6] Vallittu P.K., Lassila V.P., Lappalainen Niom R. (1996). The effect of notch shape and self-cured acrylic resin repair on the fatigue resistance of an acrylic resin denture base. J. Oral Rehabil..

[7] Ganzarolli S.M., de Mello J.A., Shinkai R.S., del Bel Cury A.A. (2007). Internal adaptation and some physical properties of methacrylate-based denture base resins polymerized by different techniques. J. Biomed. Mater. Res. Part B Appl. Biomater..

[8] Lamb D.J., Ellis B., van Noort R. (1985). The fracture topography of acrylic dentures broken in service. Biomaterials.

[9] Kim S.H., Watts D.C. (2004). The effect of reinforcement with woven E-glass fibers on the impact strength of complete dentures fabricated with high-impact acrylic resin. J. Prosthet. Dent..

[10] Nejatian T., Johnson A., van Noort R. (2006). Reinforcement of denture base resin. Adv. Sci. Technol..

[11] Hedzelek W., Gajdus P. (2006). Comparison of mechanical strength of palatal denture bases made from various plastic materials. Int. J. Prosthodont..

[12] Stafford G.D., Huggett R., MacGregor A.R., Graham J. (1986). The use of nylon as a denture-base material. J. Dent..

[13] Yunus N., Rashid A.A., Azmi L.L., Abu-Hassan M.I. (2005). Some flexural properties of a nylon denture base polymer. J. Oral Rehabil..

[14] Radford D.R., Juszczyk A.S., Clark R.K.F. (2014). The bond between acrylic resin denture teeth and the denture base: Recommendations for best practice. Br. Dent. J..

[15] Praveen B., Babaji H.V., Prasanna B.G., Rajalbandi S.K., Shreeharsha T.V., Prashant G.M. (2014). Comparison of Impact Strength and Fracture Morphology of Different Heat Cure Denture Acrylic Resins: An *In vitro* Study. J. Int. Oral Health.

[16] Kanchanavasita W., Anstice H.M., Pearson G.J. (1998). Long-term flexural strengths of resin-modified glass-ionomer cements. Biomaterials.

[17] Higgs W.A.J., Lucksanasombool P., Higgs R.J.E.D., Swain M.V. (2001). Evaluating acrylic and glass-ionomer cement strength using the biaxial flexure test. Biomaterials.

[18] Wagner W.C., Chu T.M. (1996). Biaxial flexural strength and indentation fracture toughness of three new dental core ceramics. J. Prosthet. Dent..

[19] Wen M.Y., Mueller H.J., Chai J., Wozniak W.T. (1999). Comparative mechanical property characterization of 3 all-ceramic core materials. Int. J. Prosthodont..

[20] Yilmaz H., Aydin C., Gul B.E. (2007). Flexural strength and fracture toughness of dental core ceramics. J. Prosthet. Dent..

[21] Pick B., Meira J.B.C., Driemeier L., Braga R.R. (2010). A critical view on biaxial and short-beam uniaxial flexural strength tests applied to resin composites using Weibull, fractographic and finite element analyses. Dent. Mater..

[22] O’Brien W.J. (2002). Dental Materials and Their Selection.

[23] Uzun G., Hersek N. (2002). Comparison of the fracture resistance of six denture base acrylic resins. J. Biomater. Appl..

[24] Gharechahi J., Asadzadeh N., Shahabian F., Gharechahi M. (2014). Flexural strength of acrylic resin denture bases processed by two different methods. J. Dent. Res. Dent. Clin. Dent. Prospect..

[25] Doǧan A., Bek B., Çevik N.N., Usanmaz A. (1995). The effect of preparation conditions of acrylic denture base materials on the level of residual monomer, mechanical properties and water absorption. J. Dent..

[26] Honorez P., Catalan A., Angnes U., Grimonster J. (1989). The effect of three processing cycles on some physical and chemical properties of a heat-cured acrylic resin. J. Prosthet. Dent..

[27] Wright D.D., Lautenschlager E.P. (1999). The effect of processing temperature and time on the structure and fracture characteristics of self-reinforced composite poly(methyl methacrylate). J. Mater. Sci. Mater. Med..

[28] Hayhurst L., Johnson A. (2004). The effect of resin type, packing and processing methods on the Biaxial Flexural strength of polymethyl methacrylate denture base materials. Quintessence J. Dent. Technol..

[29] Piddock V., Marquis P.M., Wilson H.J. (1986). Comparison of the strengths of aluminous porcelain fired on to platinum and palladium foils. J. Oral Rehabil..

[30] Tan F., Qiao X.L., Chen J.G., Wang H.S. (2005). Effects of coupling agents on the properties of epoxy-based electrically conductive adhesives. Int. J. Adhes. Adhes..

[31] Li A.G., Kobayashi A.S., Bradt R.C. (1989). Indentation fracture toughness of sintered silicon carbide in the palmqvist crack regime. J. Am. Ceram. Soc..

